# Probing the Putative Active Site of YjdL: An Unusual Proton-Coupled Oligopeptide Transporter from *E. coli*

**DOI:** 10.1371/journal.pone.0047780

**Published:** 2012-10-22

**Authors:** Johanne Mørch Jensen, Fouzia Ismat, Gerda Szakonyi, Moazur Rahman, Osman Mirza

**Affiliations:** 1 Department of Drug Design and Pharmacology, Faculty of Health and Medical Sciences, University of Copenhagen, Copenhagen, Denmark; 2 Health Biotechnology Division, National Institute for Biotechnology and Genetic Engineering, Faisalabad, Pakistan; 3 Institute of Membrane and Systems Biology, University of Leeds, Leeds, United Kingdom; National Research Council of Italy, Italy

## Abstract

YjdL from *E. coli* is an unusual proton-coupled oligopeptide transporter (POT). Unlike prototypical POTs, dipeptides are preferred over tripeptides, in particular dipeptides with a positively charged C-terminal residue. To further understand this difference in peptide specificity, the sequences of YjdL and YdgR, a prototypical *E. coli* POT, were compared in light of the crystal structure of a POT from *Shewanella oneidensis*. Several residues found in the putative active site were mutated and the activities of the mutated variants were assessed in terms of substrate uptake assays, and changes in specificity in terms of uptake inhibition. Most strikingly, changing the YjdL specific Asp392 to the conserved Ser in YjdL obliterated the preference for a positively charged C-terminal residue. Based on this unique finding and previously published results indicating that the dipeptide N-terminus may interact with Glu388, a preliminary orientation model of a dipeptide in the YjdL cavity is presented. Single site mutations of particularly Ala281 and Trp278 support the presented orientation. A dipeptide bound in the cavity of YjdL appears to be oriented such that the N-terminal side chain protrudes into a sub pocket that opens towards the extracellular space. The C-terminal side chain faces in the opposite direction into a sub pocket that faces the cytoplasm. These data indicated a stabilizing effect on a bulky N-terminal residue by an Ala281Phe variant and on the dipeptide backbone by Trp278. In the presented orientation model, Tyr25 and Tyr58 both appear to be in proximity of the dipeptide backbone while Lys117 appears to be in proximity of the peptide C-terminus. Mutational studies of these conserved residues highlight their functional importance.

## Introduction

In bacteria peptide transport plays an important role in providing nutrients in the form of amino acids for the metabolic machinery of the cell. Peptide uptake is also involved in signaling processes by regulation of gene expression in proteolytic processes, sporulation, chemotaxis etc. [1]. In *Escherichia coli* peptide uptake is mediated by three distinct peptide transport systems: the dipeptide permeases (Dpp), the tripeptide permeases (Tpp), and the oligopeptide permeases (Opp). Two of these systems, the Dpp and Opp, are primary active transporters driven by hydrolysis of ATP and belong to the large family of ATP-binding cassette (ABC)-transporters. Tpp on the other hand, belong to the group of secondary active transporters that drive the inward translocation of di- and tripeptides by aid of the proton motive force and are found in a range of organisms spanning from bacteria to mammals. The Tpps are generally known as the proton-coupled oligopeptide transporters (POTs) and belong to the major facilitator superfamily (MFS) [2].

**Figure 1:**
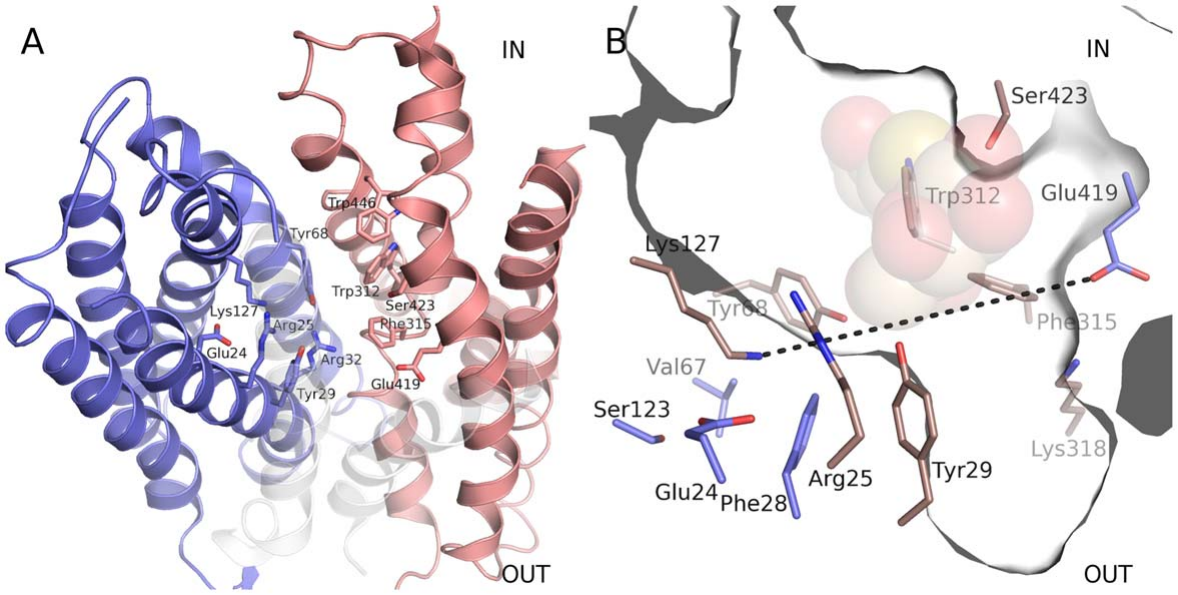
The putative peptide binding cavity. (A) View of the PepTSo structure (PDB ID: 2XUT). The N-terminal and C-terminal domains are colored blue and red, respectively, and amino acids constituting the putative peptide binding site are represented as sticks. Helix VII and VIII are semi transparent for better overview of the putative binding site. (B) View of the putative peptide binding cavity of PepTSo. Residues mutated in this study have been colored brown. The cavity shape is shown as a thin slab centered on atoms Ser423 OG, Lys127 NZ, and Glu419 OE2. TDG from the lactose permease structure (PDB ID: 1PV7) is shown as transparent spheres. The dotted line marks the division of the cavity into two pockets.

POTs are in general low affinity/high capacity transporters with a very broad range of potential di- and tripeptide substrates (apparent affinity in the micro- to millimolar range). Two POT crystals structure, from the prokaryotes *Shewanella oneidensis* (PepT_So_) and *Streptococcus thermophilus* (PepT_St_) have so far been determined [3], [4]. The structures revealed an overall fold of 12 core transmembrane helices arranged in two six-helix bundles as observed for the MFS transporters lactose permease LacY, the multidrug transporter EmrD, the fucose transporter FucP, and the glycerol 3-phosphate transporter GlpT [5], [6], [7], [8]. During the translocation mechanism the MFS transporters undergo structural changes in three major steps from outward to occluded to an inward facing conformation. The structure of PepT_So_ shows an overall inward facing occluded conformation (Figure 1A), whereas PepT_St_ display the same overall conformation, however, entirely open toward the cytoplasm. A number of key amino acid residues affecting peptide selectivity and transport have been investigated by mutational analyses [9], [10], [11], [12], [13], [14], [15], [16], [17], [18]. These studies have mainly focused on the human intestinal POT hPepT1 that, besides transporting di- and tripeptides derived from food, transports peptide-like drugs such as β-lactam antibiotics. The putative active site of PepT_So_ harbors several of the investigated residues, many of which are conserved, and believed to be involved in substrate and proton transport. As most MFS members are able to facilitate bidirectional substrate translocation down a concentration gradient, it is generally believed that the ligand-binding site is accessible and able to bind substrate both in the inward and outward facing conformation.

In *E. coli* four POT members have been identified, which cluster in pairs of YdgR and YhiP (51% identity) and YjdL and YbgH (56% identity) with approximately 26–28% sequence identity between the two groups. While YdgR and YhiP are prototypic POTs transporting a range of di- and tripeptides [19], [20], YjdL and YbgH have an unusual preference for dipeptides over tripeptides specifically dipeptides with a C-terminal Lys residue [21], [22]. In contrast to YdgR where Ala-Ala appears to be one of the dipeptides with the highest relative affinity [20], a number of dipeptides e.g. Tyr-Ala, Ala-Gln, and Ala-Lys show higher relative affinities in YjdL [20]. YjdL thus seems to be dependent on the peptide side chains for relative affinity while YdgR appears to have stronger interactions with the peptide backbone.

In the absence of a crystal structure of a POT/peptide complex it is still largely unclear where and how the peptide binds. Assuming that the overall dipeptide orientation and conformation are conserved throughout the POTs, investigations of the determinants of peptide specificity between the two *E. coli* subgroups could provide valuable information in this regard. The PepT_So_ crystal structure provides an excellent starting point in search for the structural determinants of YjdL specificity compared to YdgR.

We have examined several residues by site-directed mutagenesis and functional assays of YjdL and YdgR to probe the functional differences between YjdL and YdgR. These results pinpoint important novel determinants for substrate uptake and dipeptide specificity in YjdL. Most importantly, a single YjdL specific Asp to Ser mutation was able to dramatically reduce uptake activity, eradicating the YjdL C-terminal lysyl preference. Based on our results we propose a model for the orientation of a dipeptide inside the central cavity of YjdL.

## Results and Discussion

### Identifying Potential Specificity Determining Residues in YjdL

The PepT_So_ cavity can be divided into two joined pockets, the outward and inward facing pockets, by the highly conserved opposite charges Lys127 and Glu419 (Lys117 and Glu388 in YjdL and Lys130 and Glu396 in YdgR) located on opposite sides of the cavity (Figure 1B). Superimposing the LacY/β-D-1-thiogalactopyranoside (TDG) complex structure onto PepT_So_ places the lactose analogue TDG in the inwards facing pocket between the Lys127 and Glu419, potentially indicating the substrate binding region in PepT_So_. In order to identify candidate residues determining the YjdL ligand specificity, the cavity lining residues of PepT_So_ in proximity of the superimposed TDG were identified, and the corresponding residues in YjdL and YdgR were compared (the full alignment between the POTs is presented in Figure S1).

**Figure 2:**
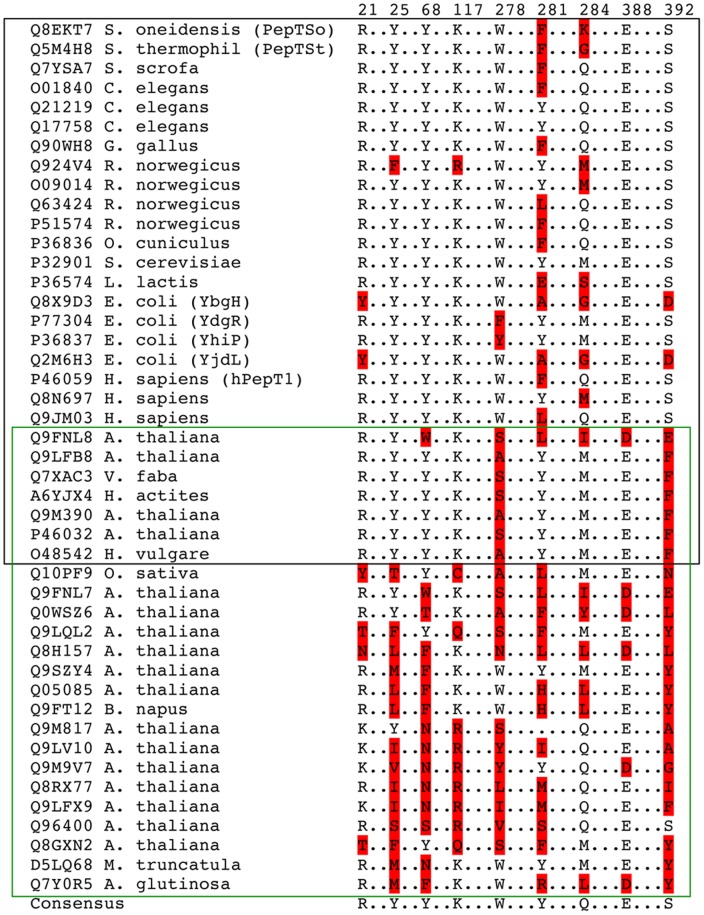
Partial sequence alignment of experimentally verified active members (Uniprot accession codes) of the POT family showing the regions of mutations. The numbering corresponds to YjdL residues. Sequences boxed in black belong to bacterial and eukaryotic POTs, while those boxed in green belong to plant POTs. Residues deviating from the POT consensus are highlighted by a red background.

The most prominent differences between YjdL and YdgR were found on positions Tyr21, Ala281, Gly284 and Asp392 (YjdL notation). The POT consensus on position 21 is Arg or Lys based on an alignment that only contains experimentally tested POTs (Figure 2). Hence a loss of charge and potential hydrogen bonding possibilities are observed in YjdL with respect to this position. Ala281 and Gly284 are found to be much bulkier residues in POTs in general (Phe or Met, respectively, Phe312 and Lys318 in PepT_So_). These positions may play a role in defining the shape and volume of the binding cavity and may thus account for the unusual ligand specificity of YjdL, in particular between di- and tripeptides. Another residue that might be involved in determining the YjdL specificity is Trp278, which is conserved in most bacterial and eukaryotic POTs but is a Tyr in YdgR. Asp392 (Ser423 in PepT_So_) on the other hand is strictly found to be a Ser in bacterial and eukaryotic POTs, however, this residue is changed to a variety of different amino acids in plant POTs (Figure 2). The presence of an YjdL specific acidic residue at this position is particularly intriguing as the strong preference of YjdL for a positively charged C-terminus indicates the presence of a counter ion in the binding cavity.

To our knowledge the role of the highly conserved Lys117 or corresponding Lys residues in the POTs has not yet been determined. In contrast, several studies have emphasized the importance of a negative charge in position 419 [11], [16], [17]. Lys117 (Lys127 in PepT_So_) is flanked by the highly conserved Glu20 and two semi conserved residues Tyr25 and 58 (29 and 68 in PepT_So_, respectively). Tyr25 and Tyr58 appear to be found almost exclusively in POT members with proven peptide preference including YdgR. A sub group of POT members, found only in plants, is involved in uptake of nitrate, chlorate, auxin, and dicarboxylates but not peptides (reviewed in [23]). In these non-peptide transporting POTs, Tyr25 and Tyr58 are not conserved (Figure 2). The hydroxyl groups of both residues protrude out towards the middle of the cavity (Figure 1B). It could therefore be speculated that one or both of these residues are involved in conserved interactions with the substrate peptide presumably through the peptide backbone.

To probe the possible roles of the above-mentioned residues, single site mutations were made in YjdL and YdgR. Using β-Ala-Lys(AMCA), a common YjdL and YdgR substrate for uptake experiments, allowed for better comparison of results obtained on mutation on corresponding positions. Initially, attempts were made to obtain reliable K_t_ values for the mutated variants. However, due to low apparent affinity of β-Ala-Lys(AMCA) for YjdL (WT K_t_ of appr. 5 mM) a determination of K_t_ was not possible (data not shown). Instead, we discuss the mutations in light of single time point and single concentration uptake experiments. Importantly, to obtain a pattern of possible ligand specificity changing mutations, uptake inhibition experiments with peptides of different properties were performed. For YjdL a “minimal” dipeptide Ala-Ala, a tripeptide Ala-Ala-Ala, a dipeptide with a bulky N-terminal side chain Tyr-Ala, a dipeptide with a bulky C-terminal side chain Ala-Gln, and a dipeptide with a positive C-terminus Ala-Lys, were used. For YdgR variants only Ala-Ala was used. All uptake assays were performed at pH 6.5 as both YdgR (Figure S2) and YjdL [17] showed the highest uptake level at this bulk pH.

### Lys117 is Important for YjdL but Essential for YdgR Function

**Figure 3:**
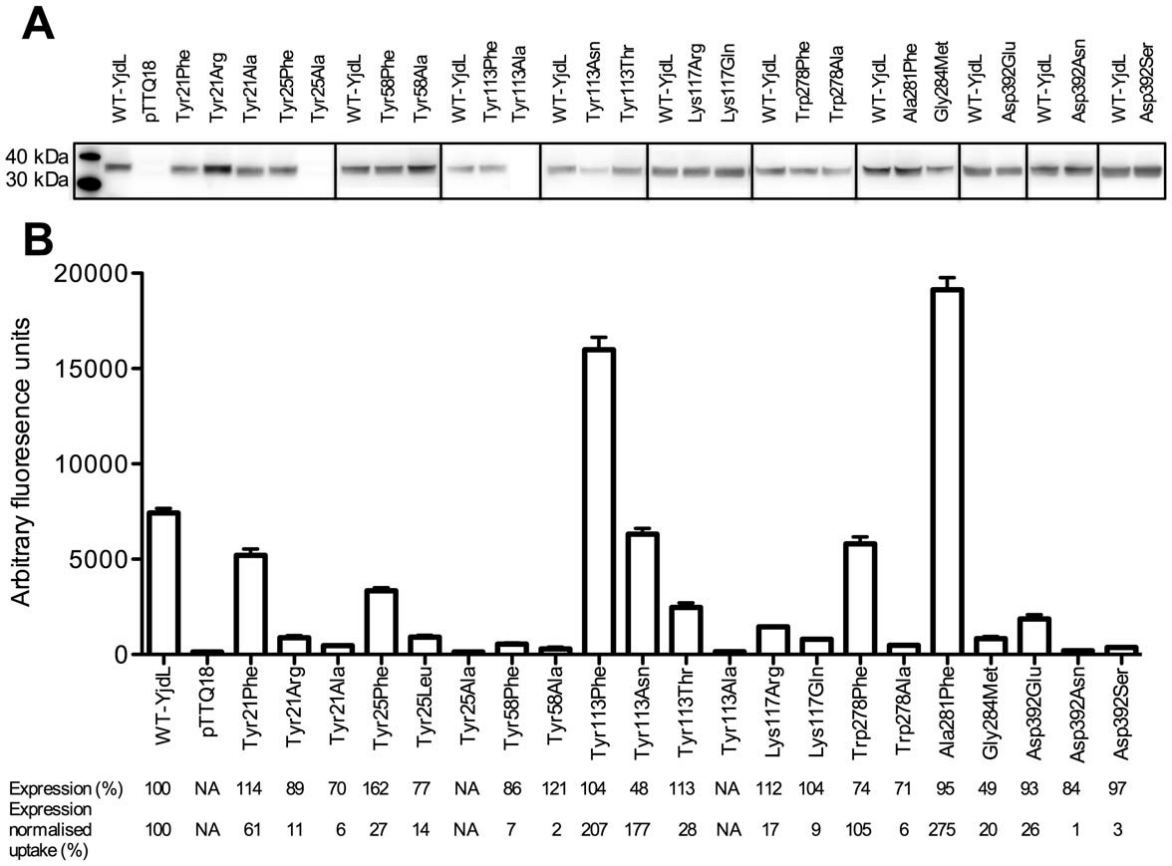
functional analyses of YjdL variants. (A) Representative western blots of YjdL mutants. (B) β-Ala-Lys(AMCA) uptake (0.2 mM, 5 min) by YjdL mutants in uptake buffer, pH 6.5. Error bars indicate SEM (n ≥3). Tyr25Ala, Tyr58Ala, and Tyr113Ala are not significantly different, P<0.05, from background levels (pTTQ18).

Lys117 of YjdL is highly conserved within the peptide-translocating POTs and in the non peptide-translocating POTs, which also contain Arg at this position (Figure 2). Although regarded as a conservative mutation, the uptake level for the YjdL Lys117Arg was surprisingly low (17%) and close to the uptake level of Lys117Gln (9%) (Figure 3). Even more dramatic, the same changes of Lys130 in YdgR led to complete lack of substrate uptake, although the expression levels for these variants were at WT-YdgR level. Similarly, mutation of this same residue in PepT_St_ resulted in complete lack of uptake [4]. Thus a positive charge and size similarity at this position does not retain activity in general, although YjdL seems to tolerate changes better than YdgR and PepT_St_.

In PepT_So_ Lys127, localized in the inward facing pocket, is tightly surrounded (less than 5 Å distance) by Glu24, Arg25, Phe28, Val67, Tyr68 and Ser123. Accommodating an Arg at position 127 at a similar conformation would require substantial conformational changes of the surrounding residues. In YdgR all but Ser123 (Asn126) are conserved. Therefore an explanation to Arg incompatibility could be that the immediate environment would restrict an Arg in a conformation unsuitable for function. In YjdL Ser123 is changed to Tyr113 and Arg25 to Tyr21. The latter could alter the cavity locally, possibly leaving more conformational space for an Arg, thereby potentially explaining the difference in activity compared to YdgR. Interestingly, YjdL does not accommodate an Arg at position 21 as uptake is severely hampered (11%) (Figure 3). In contrast, changing Tyr113 to a Phe or Asn, as found in YdgR, strikingly increases uptake almost 2-folds (Figure 3). These mutations emphasize the functional role of the region surrounding Lys117, which was also indicated by previous studies on Glu20 [17].

All natural POT substrates reported to date are negatively charged or contain a negatively charged group, suggesting the existence of a highly conserved positive counter ion. In case of dipeptides it has been shown that both the C- and N-terminus are essential for translocation, however, apparently only the N-terminus is important for affinity [24]. Our investigation of Lys117 and Lys130 in YjdL and YdgR, respectively, emphasizes the importance of this Lys. We speculate that this residue could be involved in interacting with the C-terminus.

### The Two Semi-conserved Tyr residues are Important for Substrate Uptake in YjdL and YdgR

**Figure 4:**
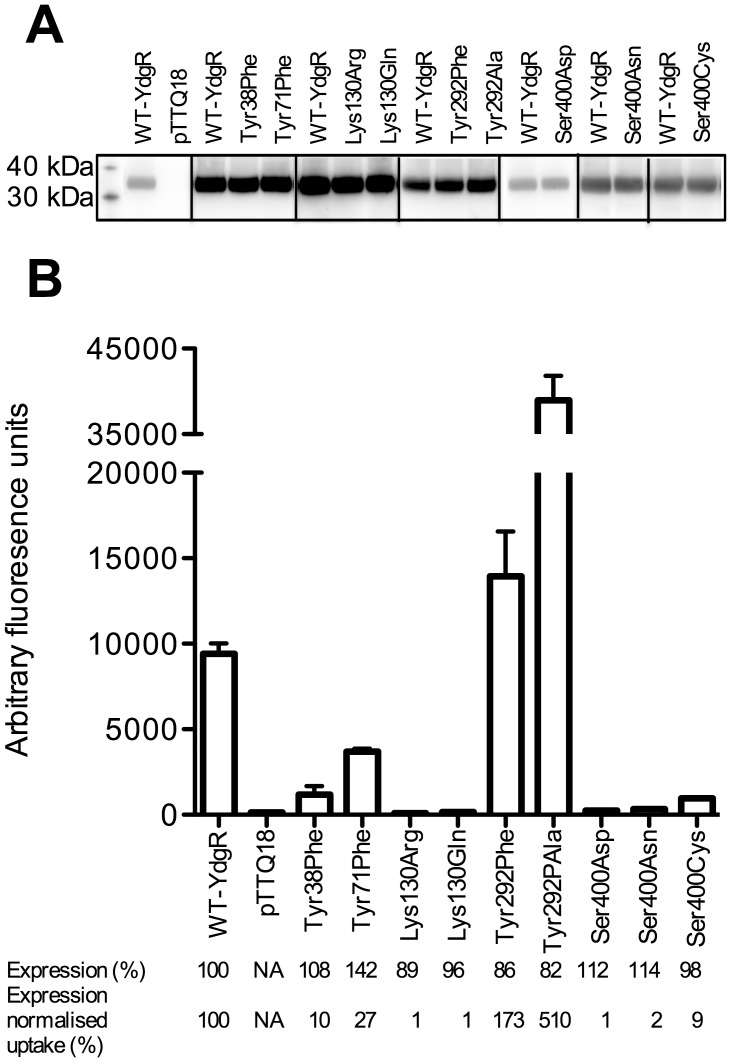
functional analyses of YdgR variants. (A) Representative western blots of YdgR mutants. (B) β-Ala-Lys(AMCA) uptake (0.2 mM, 5 min) by YdgR mutants in uptake buffer, pH 6.5. Error bars indicate SEM (n ≥3). Lys133Arg and Lys133Gln are not significantly different, P>0.05, from background levels (pTTQ18).

Mutating Tyr25 to Phe reduced uptake to 27% and further to 14% upon mutation to Leu (Figure 3). The Tyr25Ala variant failed to express. Furthermore, changing Tyr38 (corresponding to Tyr25) to Phe in YdgR significantly reduced uptake (10%) (Figure 4). Mutations corresponding to this Tyr residue have previously been reported in rabbit PepT1 where a double mutant in the rabbit PepT1 Tyr30Phe/Tyr31Phe (Phe24/Tyr25 in YjdL) showed no transport activity [15] and in PepT_St_ where the Tyr29Phe change was suggested to have an effect on affinity and selectivity of the tested peptides [4].

Mutation of the second semi-conserved residue Tyr58 to Phe resulted in a prominent reduction in uptake (7%) (Figure 3). Tyr58Ala expressed to WT levels, however, uptake significant from background levels could not be measured. In YdgR the corresponding mutation Tyr71Phe also led to a significant reduction in uptake (27%) (Figure 4). Previous mutational studies of the residue have been contradictory. Studies on rabbit PepT1 Tyr64 (Tyr58 in YjdL) was mutated to Phe and showed uptake similar to WT [15]. In contrast to this it has been reported that no transport current was detectable for neutral dipeptides in *Xenopus oocytes* expressing rabbit PepT1 Tyr64Phe [12].

The studies presented here on YdgR and YjdL show that the hydroxyl group of both Tyr residues are important for substrate uptake. It should be kept in mind that the importance of these residues may differ from one substrate to another, which highlights the importance of using the same substrate for comparing two different transporters.

Interestingly, for Tyr25Phe an excess of 10-fold decrease in relative affinity (in terms of IC_50_ values) of Ala-Ala, Tyr-Ala, and Ala-Gln was observed whereas Ala-Lys remained unchanged compared to WT-YjdL (Table 1, Figure S3). For Tyr58Phe the relative affinities for Ala-Ala, Tyr-Ala, and Ala-Lys decreased more than 10-fold compared to WT-YjdL, whereas that of Ala-Gln change to a lesser extent (4-fold, Table 1). In sharp contrast to YjdL, the decrease in uptake of YdgR Tyr38Phe and Tyr71Phe was not accompanied by an increase in the Ala-Ala IC_50_ value, which remained at a WT-YdgR level (Table 1). Similar results were observed for Ala-Ala in PepT_St_ studies [4].

**Table 1:** Primers used for site-directed mutagenesis.

Mutant	Forward primer sequence	
Tyr21Phe	5′ TCCAAATCTGGGAG**TTT**TTCAGTTTTTACGGC 3′	
Tyr21Ala	5′ TCCAAATCTGGGAG**GCG**TTCAGTTTTTACGGC 3′	
Tyr25Phe	5′ TACTTCAGTTTT**TTT**GGCATGCGTGCCTTA 3′	
Tyr25Leu	5′ CAAATCTGGGAGTACTTCAGTTTT**TTA**GGCATGCGTGCCT 3′	
Tyr25Ala	5′ TACTTCAGTTTT**GCG**GGCATGCGTGCCTTA 3′	
Tyr58Phe	5′ GCTTCTCTGGTT**TTT**GTTACCCCTAT 3′	
Tyr58Ala	5′ GCTTCTCTGGTT**GCG**GTTACCCCTAT 3′	
Tyr113Phe	5′ ATTATTTGTGGC**TTT**GGTTTATTCAAA 3′	
Tyr113Asn	5′ GCTGGCAATCATTATTTGTGGC**AAC**GGTTTATTCAAATCAAACAT 3′	
Tyr113Thr	5′ GCTGGCAATCATTATTTGTGGC**ACC**GGTTTATTCAAATCAAACAT 3′	
Tyr113Ala	5′ ATTATTTGTGGC**GCG**GGTTTATTCAAA 3′	
Trp278Ala	5′TCGGGACATTGTTC**GCG**GTACTGGCACAAC 3′	

IC_50_ values (mM) with SEM (n ≥3) of selected dipeptides tested on YjdL and YdgR variants.

a IC_50_ values determined at 0.2 mM β-Ala-Lys(AMCA) and 5 min incubation.

b IC_50_ values determined at 0.5 mM β-Ala-Lys(AMCA) and 15 min incubation.

Taken together these observations imply that prominent differences in dipeptide-backbone interactions exist between YdgR and PepT_St,_ representing a prototypical binding mode, and YjdL. Both Tyr residues are highly conserved among peptide translocating POTs and point out towards the cavity. Therefore the presented results could indicate that these residues are involved in interactions with the peptide backbone. In comparison for example, the relative affinity of the above dipeptides was unchanged for the unconserved Tyr113Phe and 2-fold or less for Tyr21Phe (Table 1).

### Trp278Phe Selectively Decreases the Relative Affinity for Ala-Ala

Changing Trp278 to Phe had no apparent effect on uptake while a change to Ala abolished uptake (Figure 3). It was somewhat surprising that the relative affinity of Ala-Ala decreased 4-folds while those of Tyr-Ala, Ala-Gln and Ala-Lys remained unchanged compared to WT-YjdL (Table 1). Thus apparently this YdgR mimicking mutation in YjdL selectively weakens Ala-Ala affinity. In Tyr-Ala, Ala-Gln, or Ala-Lys the destabilizing effect of the Trp278Phe on the “Ala-Ala” part of the dipeptide could presumably be overcome by favorable side chain interactions. Other studies have shown that mutating the residue corresponding to Trp278 in YdgR (Phe289) to Ser or Leu result in complete lack of uptake of β-Ala-Lys(AMCA) [18]. Mutating Trp294, the corresponding residue in hPepT1, to Ala resulted in a significant increase of K_t_ and decrease of V_max_ for Gly-Sar [11]. These results are in accord with the results presented on Trp278 here. Cumulated these observations suggest interactions between Trp278 and the dipeptide, presumably backbone.

### The Outward Facing Pocket could Harbor the N-terminal Side Chain

Ala281 and Gly284 were mutated to Met and Phe, respectively, in order to assess whether incorporation of a POT consensus residue at these positions could change the specificity of YjdL. Uptake by Ala281Phe increased almost 3-fold compared to WT-YjdL (Figure 3). The reverse mutation in YdgR, Tyr292Ala surprisingly showed an almost 4-fold increase in uptake (Figure 4). Thus, at the same position, a volume increasing mutation in YdgR has the same effect on uptake as a volume decreasing mutation in YjdL.

Two observations in YjdL indicated that an N-terminal Tyr residue confers relative affinity to the dipeptide: first, the Trp278Phe mutation only decreased the relative affinity of Ala-Ala but not Tyr-Ala. Secondly, inclusion of Tris in the uptake assay increased the IC_50_ values of Ala-Ala but not Tyr-Ala due to Tris inhibition of YjdL [21] (Figure S4) although the relative affinities of YjdL for Ala-Ala and Tyr-Ala were not significantly different in MES buffer, pH 6.5 (Table 1).

To understand this in greater details we probed the relative affinity of the dipeptides with a C-terminal Lys residue: Met-Lys, Tyr-Lys, and Trp-Lys for WT-YjdL (Table 2). Introduction of the above residues increased the IC_50_ values approximately 2-fold compared to the Ala-Lys value. The same trend was observed for Tyr25Phe (Table 2). However, for Ala281Phe the IC_50_ values were found to decrease up to approximately 10-fold for Tyr-Lys compared to Ala-Lys. Similar trends were observed with Met-Lys and Trp-Lys (Table 2). These results imply that the phenyl group of Ala281Phe may be involved in interactions with the bulkier residue at the N-terminal position of the dipeptide leading to a gain in relative affinity. Therefore we speculate that Ala281 could be localized in or near a region that harbors the N-terminal side chain. This affinity increasing effect is only observed when Lys is present at the C-terminus, indicating the importance of a Lys for anchoring the dipeptide (Table 2).

**Table 2:** IC_50_ values of selected dipeptides on YjdL and YdgR variants.

IC_50_ (mM)	Ala-Ala	Tyr-Ala	Ala-Gln
*YjdL*			
WT-YjdL^a^	2.8±0.6	2.4±0.4	0.6±0.1
Tyr21Phe^a^	4.5±0.3	4.7±0.8	1.1±0.1
Tyr25Phe^a^	>50	33.7±3.9	36.1±5.4
Tyr113Phe^a^	1.9±0.1	1.3±0.3	1.1±0.1
Trp278Phe^a^	9.9±1.0	2.8±0.4	0.9±0.2
Ala281Phe^a^	>50	33.2±3.4	21.7±2.2
WT-YjdL^b^	2.2±0.1	2.4±0.2	1.9±0.4
Tyr58Phe^b^	28.1±5.0	24.1±2.8	2.5±0.1
*YdgR*			
WT-YdgR^a^	0.39±0.04		
Tyr71Phe^a^	0.37±0.03		
Tyr292Phe^a^	0.15±0.03		
Tyr292Ala^a^	0.59±0.05		
WT-YdgR^b^	1.04±0.06		
Tyr38Phe^b^	0.92±0.06		

IC_50_ values (mM) with SEM (n ≥3) of C-terminal lysine dipeptides tested on YjdL variants.

a IC_50_ values determined at 0.2 mM β-Ala-Lys(AMCA) and 5 min incubation.

b IC_50_ values determined at 0.5 mM β-Ala-Lys(AMCA) and 15 min incubation.

Substrate uptake by Gly284Met was low (20% (Figure 3)) suggesting that Met is not optimal at this position. As Gly284 is located at the very bottom of the outward facing pocket (Lys318 in PepT_So_), investigations of this residue were not pursued further.

### Asp392Ser Abolishes C-terminal Lysine Preference and Increases Tripeptide Specificity

**Figure 5:**
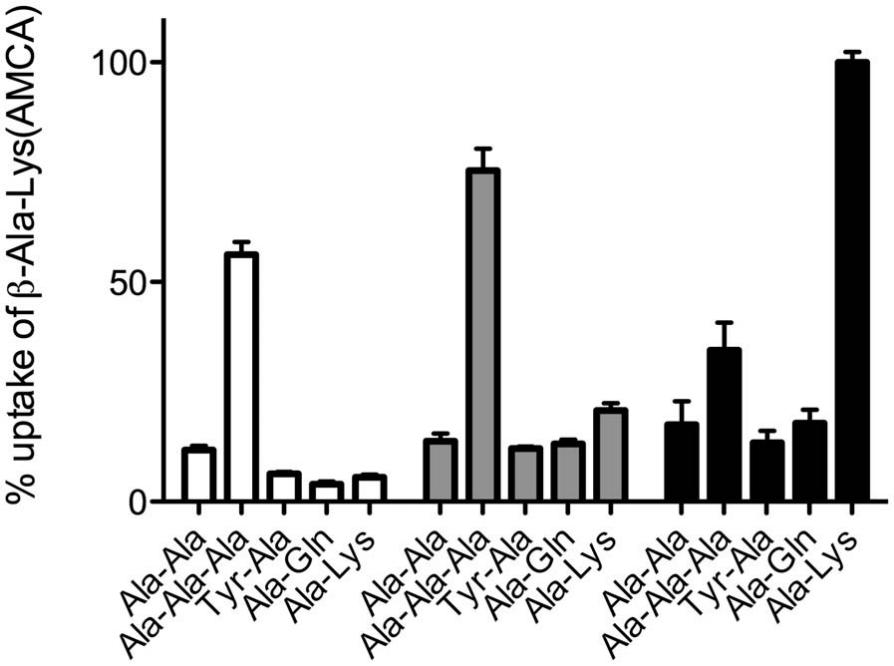
Inhibition profiles for WT-YjdL (white), Asp392Glu (grey), and Asp392Ser (black). The cells were incubated in assay buffer pH 6.5 containing 0.5 mM β-Ala-Lys(AMCA) and 50 mM ligand. Error bars indicate SEM (n ≥3).

All peptide translocating POTs contain a Ser at the Asp392 position of YjdL (located in the inward facing pocket) (Figure 1B & 2). To probe the function of this residue three mutations Asp392Glu, Asp392Asn, or Asp392Ser were introduced. The uptake level of Asp392Glu under standard conditions was 26% while those for Asp392Asn and Asp392Ser were comparable to background levels (Figure 3 and Figure S5). Inhibition profiles of WT-YjdL, Asp392Glu, and Asp392Ser were determined with 50 mM competitor (Ala-Ala, Tyr-Ala, Ala-Gln, Ala-Lys, or Ala-Ala-Ala) (Figure 5) at substrate concentration, which gave uptake levels at least 10-fold higher than the background. For WT-YjdL uptake was reduced to approximately 10% or lower by all tested peptides except for the tripeptide Ala-Ala-Ala, which was only reduced to 55%, confirming the dipeptide specificity of WT-YjdL [21]. While the inhibition profile of Asp392Glu resembled WT-YjdL, Asp392Ser showed a dramatic reduction in Ala-Lys inhibition. This clearly showed that removal of the negative charge on position 392 in YjdL abolishes the preference for a dipeptide with a positively charged C-terminal side chain, suggesting a direct interaction between these residues. Ala-Ala-Ala interestingly became more potent, inhibiting uptake to 34% in Asp392Ser implying that removing the negative charge and creating space at this position increases the preference for a tripeptide. Of all changes made in YjdL, only the Asp392 changes the di- and tripeptide balance (Figure S6). The structural reason for increased tripeptide specificity is not clear but interestingly, in the inward facing conformation of the PepT_So_ structure, Ser423 (corresponding to Asp392) is in proximity of the conserved FYING sequence motif found in POTs. In the outward facing FucP structure [7] the regions corresponding to the FYING motif and Ser423 are in close contact. Previously, mutations in the FYING motif of the yeast POT PTR2 from *S. cerevisiae* altered the balance between peptide length preferences [14]. Thus it could be speculated that the Ser mutations of Asp392 may be involved in local structural changes that shift the balance toward Ala-Ala-Ala preference. An outward facing POT crystal structure could be helpful for investigating this further.

To test whether the reverse mutation of Asp392Ser in YdgR would lead to a lowering of tripeptide preference and increase preference of a positively charged C-terminal side chain, Ser400 was mutated to Asp, Asn, and conservatively to Cys. Although expression levels were similar to WT-YdgR for all, Ser400Asp and Ser400Asn uptake was too low to enable inhibition studies. To enable peptide specificity studies of Ser400Asp/Asn, other substrates such as Gly-Sar [19] should be tested in order to obtain significant uptake.

### An YjdL Orientation Model for C-terminal Lys Dipeptides

**Figure 6:**
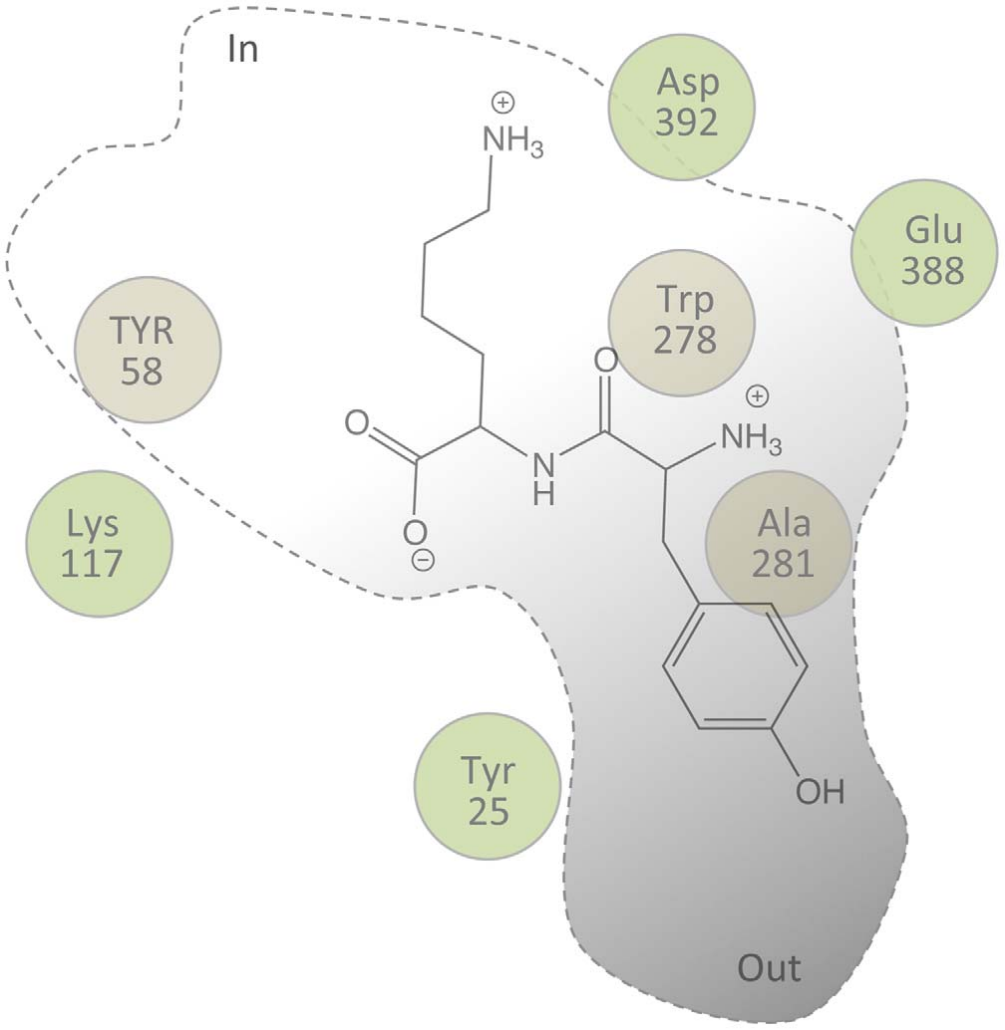
Schematic dipeptide orientation model for YjdL exemplified by Tyr-Lys. The cavity shape has been adapted from PepT_So_ (Figure 1B). The labeled spheres indicate the position of the C-alpha of the corresponding residue in PepT_So_. Residues behind the plane of the peptide are colored brown.

The identification of Asp392 in YjdL as a possible counter ion for the C-terminal Lys side chain is highly important. Here the unusual ligand preference enabled identification of an “anchoring point” for creating an Xxx-Lys dipeptide orientation model in the putative active site of YjdL. This may not have been possible with the prototypical POTs, which apparently bind the peptide backbone with high affinity, in contrast to YjdL, which depends more on the side chains, as shown by several of the data presented here. We have previously shown evidence indicating that the N-terminal of Xxx-Lys peptides could be interacting with Glu388 [17]. This information provides a second anchoring point. Third, studies on hPepT1 of have shown that dipeptides are bound and transported in a *trans* conformation [25]. Based on this cumulated information we present a schematic Tyr-Lys orientation model (Figure 6). This model is able to explain several of the observations presented here: In the trans conformation the Lys side chain protrudes into the inward facing pocket while the Tyr side chain protrudes in to the outward facing pocket. The side chains of Tyr25, Tyr58 and Trp278 are in proximity of the dipeptide backbone and the C-terminus is in close proximity to Lys117, while Ala281 is part of the outward facing pocket harboring the N-terminal side chain.

This orientation model may well be applicable to peptide translocating POTs in general, with Tyr-Lys occupying the “conserved” space in the cavity. Our model fits well with studies of the human kidney POT PepT2, which suggested an arrangement of the respective side chains of dipeptide-based inhibitors in a two-pocket model [26]. In the terminology used by Theis *et al.* the P1- and P2-pockets would correspond to the outward and inward facing pockets, respectively [26].

## Materials and Methods

### The yjdL and ydgR Expression Vector

The pTTQ18-*yjdL* expression vector contains the *yjdL* gene fused to a 3′ nucleotide linker encoding a His_6_-tag as previously described [21]. The *ydgR* gene from *E. coli* MG1655 genomic DNA was amplified using the primers 5′-GCAGAATTCGCATATGTCCACTGCAAACCAAAAACCAACTGAAAGC-3′ and 5′-CCCAAGCTTTTAATGGTGATGGTGATGGTGCGCTACGGCTGCTTTCGCCGCTTTGTCTGC-3′ in a touchdown PCR [27]. The *ydgR* gene was modified with an EcoRI site at the 5′-end and a HindIII site and oligonucleotides encoding a His_6_-tag at the 3′-end. The expression vector pTTQ18-*ydgR* was constructed by inserting the PCR product, digested with EcoRI/HindIII, into the corresponding sites of pTTQ18 [28] and the construct was verified by DNA sequencing.

### Site-directed Mutagenesis

Mutations in the *yjdL* gene were introduced using the expression vector pTTQ18-*yjdL* as a template and the QuikChange method (Stratagene). The primers for each mutagenesis reaction are shown in Table 3. The following mutants were purchased from GenScript USA: YjdL-Tyr21Arg, YjdL-Lys117Arg, YjdL-Lys117Gln, YjdL-Trp278Phe, YjdL-Ala281Phe, YjdL-Gly284Met, YjdL-Asp392Glu, YjdL-Asp392Asn, YjdL-Asp392Ser, YdgR-Tyr38Phe, YdgR-Tyr71Phe, YdgR-Lys130Arg, YdgR-Lys130Gln, YdgR-Tyr292Phe, YdgR-Tyr292Ala, YdgR-Ser400Asp, YdgR-Ser400Asn, YdgR-Ser400Cys. All mutations were confirmed by DNA sequencing.

**Table 3:** IC_50_ values of C-terminal lysyl dipeptides.

IC_50_ (mM)	Ala-Lys	Met-Lys	Tyr-Lys	Trp-Lys
WT-YjdL^a^	0.28±0.01	0.53±0.03	0.55±0.04	0.60±0.07
Tyr21Phe^a^	0.45±0.07			
Tyr25Phe^a^	0.40±0.08	0.61±0.02	0.67±0.08	0.58±0.06
Tyr113Phe^a^	0.34±0.04			
Trp278Phe^a^	0.38±0.00			
Ala281Phe^a^	0.26±0.02	0.17±0.01	0.03±0.00	0.09±0.01
WT-YjdL^b^	0.89±0.16			
Tyr58Phe^b^	>50			

Forward primer sequences used to generate mutations in *yjdL* by site-directed mutagenesis. The reverse complement of the forward primers was used as the reverse primers.

### Expression

Plasmids were transformed into *E. coli* BL21(DE3)pLysS cells by heat shock and transformants were selected on agar plates containing 100 µg/mL ampicillin and 34 µg/mL chloramphenicol. Single transformants were inoculated into 2 mL LB-media with antibiotics as above and grown overnight. The cultures were diluted 1∶50 in 5 mL LB-media with antibiotics as above, and at an OD_600_ of 0.5–0.8 protein expression was induced by the addition of 1 mM isopropyl β-D-1-thiogalactopyranoside (IPTG). The cells were harvested after three hours of induction by centrifugation and resuspended in uptake buffer (50 mM MES pH 6.5, 140 mM NaCl, 5.4 mM KCl, 1.8 mM CaCl_2_, 0.8 mM MgSO_4_, and 5 mM glucose).

### Western Blots

Western blots were performed essentially as previously described [17]. Briefly, pellets from 0.25 mL of cell suspensions (OD_600_ = 10) were resuspended in lysis buffer containing 50 mM Tris-HCl pH 7.5, 150 mM NaCl, 1% Triton X-100 and one Complete Protease Inhibitor Cocktail tablet (Roche)/10 mL buffer and incubated 30 min on ice. The samples were sonicated for 15 sec followed by centrifugation (12,000 *g*, 15 min, 4°C) and the cleared lysates, which contain the inner membranes, were separated by SDS-PAGE and subsequently blotted onto a PVDF membrane using an XCell II module (Invitrogen). Immunodetection was performed using mouse anti-His_6_ and HRP-conjugated rabbit anti-mouse antibodies (IBA) followed by SuperSignal® West Pico chemiluminescent substrate (Pierce). Signals were detected using a MicroChemi imaging system. Western blots of all mutants were performed in triplicates and expression levels were determined by quantification of band intensities using ImageJ [29] and normalized to WT expression levels.

### Uptake and Inhibition Assays

Uptake assays were performed as previously described [17], [20]. In a final assay volume of 100 µL; 0.2 mM β-Ala-Lys-N-7-amino-4-methylcoumarin-3-acetic acid (β-Ala-Lys(AMCA), Biotrend) (10 µL) and various concentrations of ligands (purchased from Bachem or Sigma-Aldrich) or uptake buffer (50 µL) were incubated with bacterial cell suspension (OD_600_ = 10, 40 µL) at 37°C. After 5 min, the uptake was stopped by addition of 500 µL ice-cold uptake buffer followed by immediate centrifugation and washing twice in ice-cold uptake buffer. Finally the cell pellet was resuspended in 100 µL uptake buffer, and the uptake was quantified by fluorescence measurements (excitation at 340 nm and emission at 460 nm) on a Safire 2 fluorimeter. Non-specific uptake was measured as described above on IPTG-induced *E. coli* BL21(DE3)pLysS cells harboring the empty pTTQ18 vector. All uptake assay experiments were reproduced at least three times independently and data analyses were performed using GraphPad Prism ver. 5.

### Sequence Alignment and Structural Analyses

Protein sequences for POT members with documented peptide translocation activity were retrieved from the UNIPROT database (http://www.uniprot.org/), aligned with Clustal Omega [30] (Figure S1), and edited manually using Jalview [31]. Structure visualization and cavity analyses were performed using PYMOL (The PyMOL Molecular Graphics System, Version 1.5.0.1 Schrödinger, LLC).

## Supporting Information

Figure S1 **Full-length sequence alignment between PepT_So_, YdgR, and YjdL.**
(PDF)

Figure S2 **β-Ala-Lys(AMCA) uptake as a function of bulk pH of WT-YdgR.**
(PDF)

Figure S3 **Inhibition curves for WT-YjdL of Ala-Ala (circles), Tyr-Ala (squares), and Ala-Gln (triangles).**
(PDF)

Figure S4 **Inhibition profiles of 25 mM Tris-HCl (white) and 25 mM HEPES (gray) of WT-YjdL and WT-YdgR.**
(PDF)

Figure S5 **β-Ala-Lys(AMCA) uptake (0.5 mM, 15 min) by YjdL and YdgR mutants in uptake buffer, pH 6.5.**
(PDF)

Figure S6 **Inhibition profiles of 50 mM Ala-Ala (white) and 50 mM Ala-Ala-Ala (gray) of YjdL and YdgR mutants.**
(PDF)
