# Supplementary material for: Probing the Putative Active Site of YjdL: An Unusual Proton-Coupled Oligopeptide Transporter from E. coli
Source: PLoS One. 2012 Oct 22;7(10):e47780. doi: 10.1371/journal.pone.0047780 (PMC3478282; doi:10.1371/journal.pone.0047780)
Supplement: Figure S1 — Full-length sequence alignment between PepTSo, YdgR, and YjdL. (PDF) [file pone.0047780.s001.pdf]

Supplementary Material

Figure S1

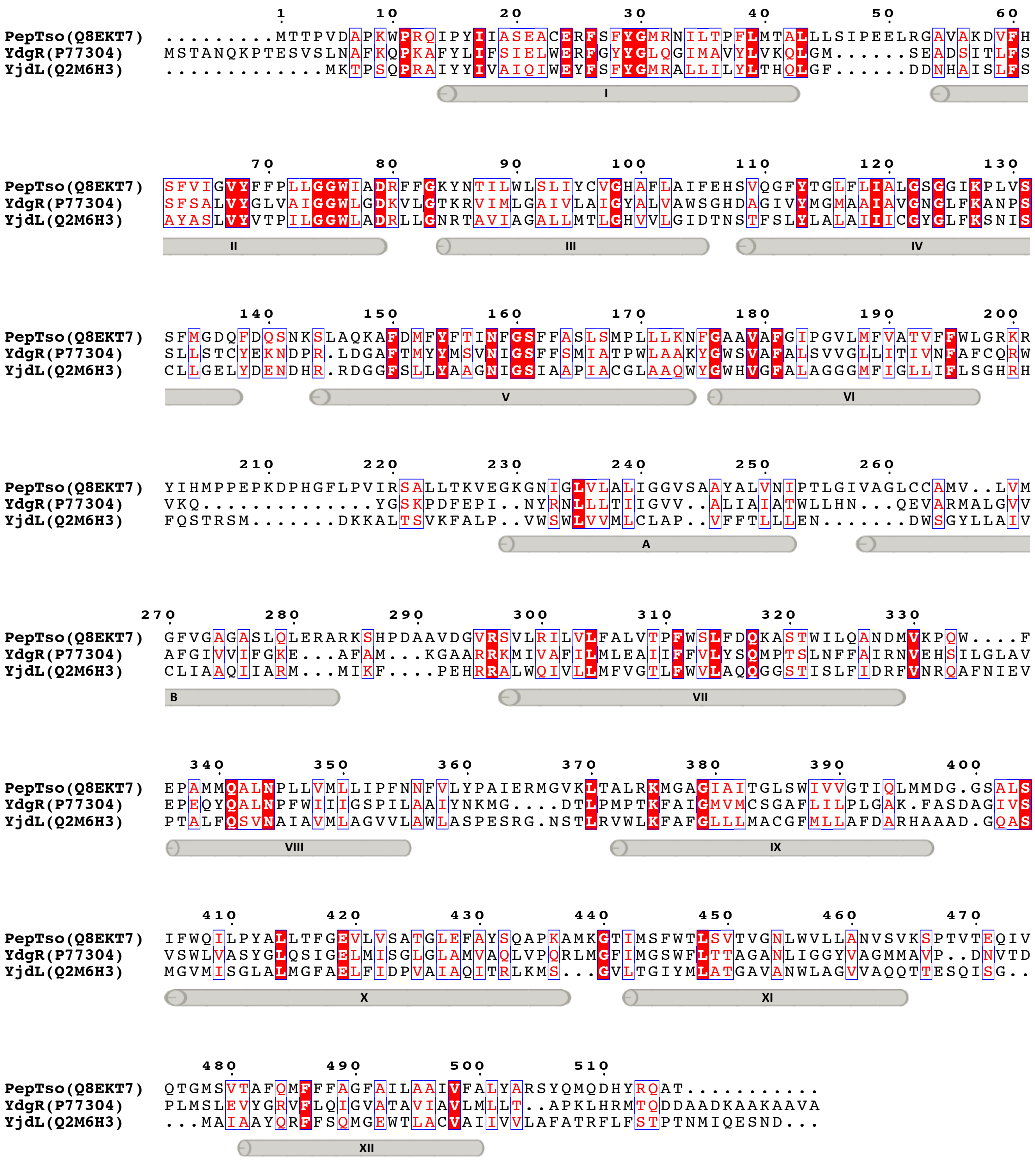

Figure S1 Full-length sequence alignment between $PepT_{So}$, YdgR and YjdL. The overall sequence identity/similarity between YjdL and PepTso was found to be 25/41% and 24/40% between YdgR and $PepT_{So}$. In comparison these values were somewhat higher for the cavity lining helices I, II, IV, V, X and XI (35/50% and 34/53%).
