# Supplementary material for: Probing the Putative Active Site of YjdL: An Unusual Proton-Coupled Oligopeptide Transporter from E. coli
Source: PLoS One. 2012 Oct 22;7(10):e47780. doi: 10.1371/journal.pone.0047780 (PMC3478282; doi:10.1371/journal.pone.0047780)
Supplement: Figure S2 — β-Ala-Lys(AMCA) uptake as a function of bulk pH of WT-YdgR. (PDF) [file pone.0047780.s002.pdf]

Figure S2

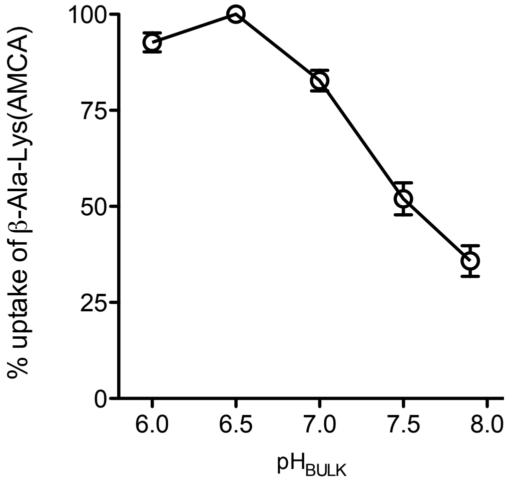

Figure S2 β-Ala-Lys(AMCA) uptake as a function of bulk pH of WT-YdgR. Cells were incubated 5 min with uptake buffer (50 mM MES or 50 mM MOPS) pH 6.0-7.9, containing 0.5 mM β-Ala-Lys(AMCA). Error bars indicate SEM ($n \geq 3$).
