# Supplementary material for: Probing the Putative Active Site of YjdL: An Unusual Proton-Coupled Oligopeptide Transporter from E. coli
Source: PLoS One. 2012 Oct 22;7(10):e47780. doi: 10.1371/journal.pone.0047780 (PMC3478282; doi:10.1371/journal.pone.0047780)
Supplement: Figure S3 — Inhibition curves for WT-YjdL of Ala-Ala (circles), Tyr-Ala (squares), and Ala-Gln (triangles). (PDF) [file pone.0047780.s003.pdf]

Figure S3

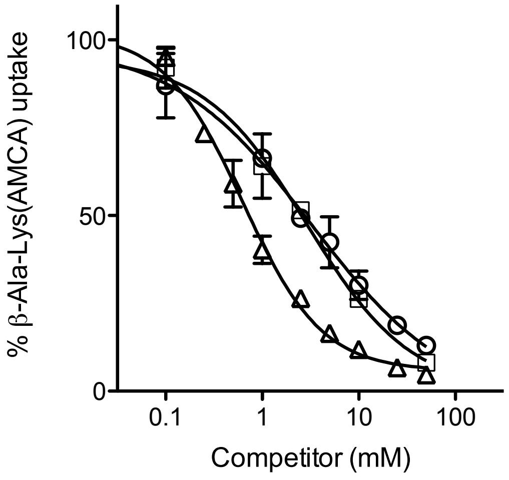

Figure S3 Inhibition curves for WT-YjdL of Ala-Ala (circles), Tyr-Ala (squares), and Ala-Gln (triangles). Cells were incubated 5 min in uptake buffer pH 6.5 containing 0.2 mM β-Ala-Lys(AMCA). Error bars indicate SEM (n ≥ 3).
