# Supplementary material for: Probing the Putative Active Site of YjdL: An Unusual Proton-Coupled Oligopeptide Transporter from E. coli
Source: PLoS One. 2012 Oct 22;7(10):e47780. doi: 10.1371/journal.pone.0047780 (PMC3478282; doi:10.1371/journal.pone.0047780)
Supplement: Figure S4 — Inhibition profiles of 25 mM Tris-HCl (white) and 25 mM HEPES (gray) of WT-YjdL and WT-YdgR. (PDF) [file pone.0047780.s004.pdf]

Figure S4

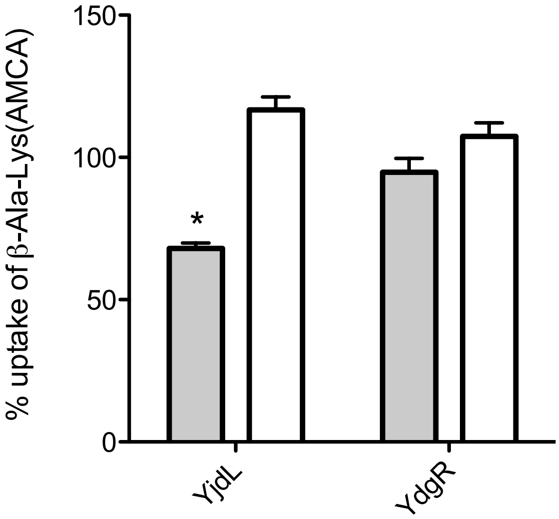

Figure S4 Inhibition profiles of 25 mM Tris-HCl (white) and 25 mM HEPES (Gray) of WT-YjdL and WT-YdgR. Cells were incubated 5 min with uptake buffer (50 mM MOPS, pH 7.4) containing 0.2 mM β-Ala-Lys(AMCA). Error bars indicate SEM ($n \geq 3$) and * indicates that values are significantly different, $P < 0.05$, from uninhibited YjdL.

Compared to previous peptide specificity studies on WT-YjdL performed at pH 7.4 (25 mM Tris/HEPES) the IC_{50} values of Ala-Ala and Ala-Gln was eight and six times lower at pH 6.5 (50 mM MES) while Tyr-Ala remained the same (Table 2). This appeared to be due to weak, however significant inhibition of YjdL by Tris as shown above. In comparison, WT-YdgR showed no significant inhibition by Tris.
