# Supplementary material for: Probing the Putative Active Site of YjdL: An Unusual Proton-Coupled Oligopeptide Transporter from E. coli
Source: PLoS One. 2012 Oct 22;7(10):e47780. doi: 10.1371/journal.pone.0047780 (PMC3478282; doi:10.1371/journal.pone.0047780)
Supplement: Figure S5 — β-Ala-Lys(AMCA) uptake (0.5 mM, 15 min) by YjdL and YdgR mutants in uptake buffer, pH 6.5. (PDF) [file pone.0047780.s005.pdf]

Figure S5

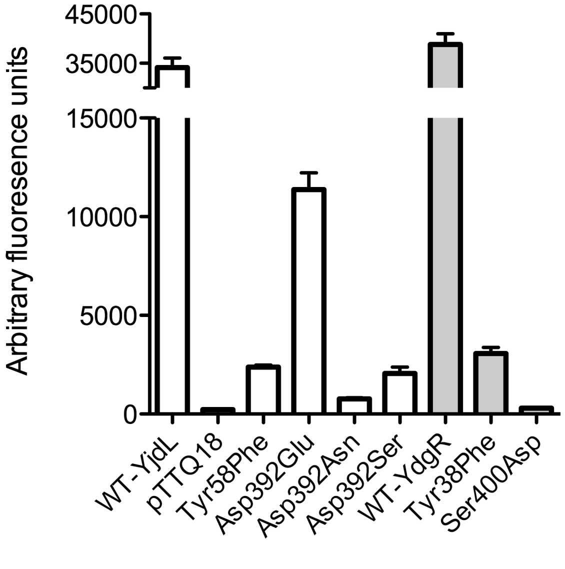

Figure S5 β-Ala-Lys(AMCA) uptake (0.5 mM, 15 min) by YjdL and YdgR mutants in uptake buffer, pH 6.5. Error bars indicate SEM ($n \geq 3$). YdgR-Ser400Asp is not significantly different, $P < 0.05$, from the background level (pTTQ18).
