# Supplementary material for: Probing the Putative Active Site of YjdL: An Unusual Proton-Coupled Oligopeptide Transporter from E. coli
Source: PLoS One. 2012 Oct 22;7(10):e47780. doi: 10.1371/journal.pone.0047780 (PMC3478282; doi:10.1371/journal.pone.0047780)
Supplement: Figure S6 — Inhibition profiles of 50 mM Ala-Ala (white) and 50 mM Ala-Ala-Ala (gray) of YjdL and YdgR mutants. (PDF) [file pone.0047780.s006.pdf]

Figure S6

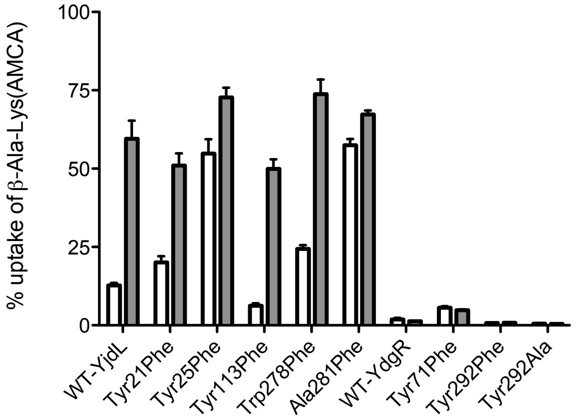

Figure S6 Inhibition profiles of 50 mM Ala-Ala (white) and 50 mM Ala-Ala-Ala (gray) of YjdL and YdgR mutants. Cells were incubated 5 min in uptake buffer pH 6.5 containing 0.2 mM β-Ala-Lys(AMCA). Error bars indicate SEM ($n \geq 3$).
